# Study on atomization mechanisms and spray fragmentation characteristics of water and emulsion butachlor

**DOI:** 10.3389/fpls.2023.1265013

**Published:** 2023-10-06

**Authors:** Wanting Yang, Wei Zhong, Weidong Jia, Mingxiong Ou, Xiang Dong, Tie Zhang, Suming Ding, Li Jiang, Xiaowen Wang

**Affiliations:** ^1^ School of Agricultural Engineering, Jiangsu University, Zhenjiang, Jiangsu, China; ^2^ Science Innovation Center, Chinese Academy of Agriculture Mechanization Sciences Group Co., Ltd., Beijing, China; ^3^ Nanjing Institute of Agricultural Mechanization, Ministry of Agriculture and Rural Affairs, Nanjing, China

**Keywords:** agriculture, pesticide, atomization fragmentation, droplet size spectrum, spray

## Abstract

Agricultural chemicals are commonly used to control pests and weeds, but cause pesticide waste problems. Oil-based emulsions are often used as pesticide formulations to improve pesticide utilization. In this study, the spray visualization experiment of the water and oil-based emulsion butachlor is carried out using an ST flat fan nozzle at 0.1–0.5 MPa pressure. The dimensionless method is used to analyze the difference in liquid sheet fragmentation morphology and disintegration process and the influence of different fragmentation methods on droplet size. It is found that the hydrophobic components in pesticide have a significant effect on the morphology and process of atomization fragmentation. When spray liquid is water, the liquid sheet breaks up into liquid ligaments due to the Rayleigh instability, then the ligaments break up into droplets. The side view of a liquid sheet is a large-amplitude wave disturbance. When the spray liquid is the emulsion butachlor, holes are generated on the liquid sheet, then the holes break up into droplets. The fragmentation method of emulsion spray is the perforation mechanism. Compared with water spray, the presence of the pesticide butachlor increases the droplet size and spray angle and improves the uniformity of droplet size distribution but reduces the breakup length. The spray angle shows a power law dependence of the Weber number with a power of 0.17 for all conditions tested here. At 0.3 MPa, DV50 increases 25%, and span decreases from 1.187 to 1.172. This study could provide reference for the addition of agricultural additives, the improvement of spray operation efficiency, and the establishment of spray fragmentation mechanism.

## 1 Introduction

At present, pesticide spraying is the most important method of modern agriculture to control crop diseases and insect pests. With the continuous progress of society and the gradual strengthening of environmental protection awareness, higher requirements have been put forward for pesticide spraying efficiency and pesticide utilization rate of plant protection machinery. How to increase the effective utilization rate of pesticides, reduce spray drift, and improve the foliar deposition has become the focus of research (Jun, 2012; Zhang et al., 2014; Wang et al., 2015).

The atomization process has a significant effect on the droplet size spectrum, and the droplet size has a very important effect on pesticide deposition and anti-drift spray (Yang et al., 2022). When the spray liquid contains oil-based emulsion, it would produce larger droplets than water spray when spraying through a flat fan nozzle, which is of great significance for controlling droplet drift (Hilz et al., 2012; Cryer and Altieri, 2017). From the early 1960s to the present, researchers have studied the influence of spray liquid characteristics on atomization (Wang et al., 2018). The decrease of surface tension in pure liquid leads to an increase in the growth rate in instability, which eventually leads to earlier liquid sheet breakup (Lefebvre and McDonell, 2017). However, studies have shown that surfactant solutions reduce surface tension and may lead to delayed breakups (Miller and Ellis, 2000). Surface tension is the most important physical property of spray liquids (Wang et al., 2018). How surface tension affects atomization fragmentation remains to be further explored. The visualization method could be used to study fragmentation physics well (Cloeter et al., 2010). The breakup length decreases with the increase of spray pressure. The appearance of hole structures on liquid sheet reduces the generation of droplets. When using emulsion-containing liquid spray, the emulsion droplets merge with the air/water interface of the liquid sheet, which enhances the disturbance in the turbulence and causes the perforation atomization (Hilz et al., 2012). In the atomization process of water and oil-in-water emulsions, the oil phase in the form of emulsions can shorten the length of the liquid sheet and expand the droplet size (Qin et al., 2010). The droplet size decreases with the increase of spray pressure (Negeed et al., 2011). Different nozzle structures affect the droplet size and velocity, and the addition of additives also affects the droplet size (Ellis and Tuck, 1999). The droplet size generated in the process of agricultural liquid atomization affects its coverage and off-target drift (Altieri and Cryer, 2018; Guler et al., 2020). Many studies have focused on the parameter of droplet volume median diameter, while the significant influence of droplet size divergence should be paid attention to in agricultural spraying.

These studies provide the basis and help for the establishment of atomization perforation regime. Although there are many studies on different liquid spray atomization mechanism, the current research on the atomization mechanism of perforation needs to be further improved. The influence of emulsion atomization mechanism on atomization quality is still a challenge (Zhao, 2012).

In this paper, the images of atomization and perforation process of water and emulsion are captured by the visualization method. The dimensionless analysis method is used to normalize the different physical properties of the spray liquid, which avoids the problem of inconsistent units of different physical properties. The spray atomization process is visualized by a high-speed camera, and the spray structure is quantitatively analyzed by image post-processing. The evolution of spray structure reflects the development of the instability of the spray liquid sheet. In addition, the effects of different atomization disturbance structures on droplet size distribution are described by measuring D_V10_, D_V50_, D_V90_, and span. The effects of different spray structures on droplet size divergence are studied. It provides a reference for the use of plant protection spray adjuvants and the improvement of pesticide utilization.

## 2 Materials and methods

### 2.1 Material and equipment

The experiment was conducted in the Key Laboratory of Modern Agricultural Equipment and Technology, Ministry of Education, Jiangsu University. The experimental temperature is ambient temperature 23°C. The spray solutions used in the experiment are water and butachlor (CAS No: 23184-66-9, Lulilai, China). The water used in the experiment is tap water, with a surface tension of 0.0724 N/m and a density of 1.019 × 10^3^ kg/m^3^. Butachlor is an oil-based emulsion pesticide, which is widely used in agricultural weed control. The concentration of butachlor in this study is 0.1%, the surface tension of this concentration is only 0.0417 N/m, and the density is 1.016 × 10^3^ kg/m^3^.

The nozzle used in the experiment is the standard flat fan spray nozzle (Lechler GmbH, Germany) produced by Lechler GmbH. The nozzle type is ST 110-01. The instruments used in the experiment are the i-speed high-speed camera (OLYMPUS, UK) produced by OLYMPUS company and the winner318 industrial spray laser particle size analyzer (Winner Particle, China) produced by Winner Particle technology Co., Ltd.

### 2.2 Experiment setup

**Figure 1:**
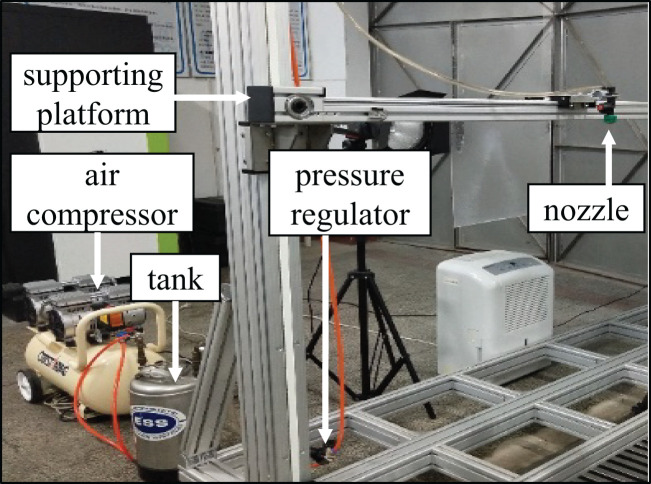
Pressure spray system includes supporting platform, air compressor, liquid tank, pressure regulator, and the spray nozzle.

The images of water and butachlor atomization and fragmentation process are captured by a high-speed camera using a Lechler flat fan nozzle under different pressures of 0.1–0.5 MPa provided by a pressure spray system as shown in **Figure 1**. The droplet size of water and butachlor is measured by a laser particle size analyzer. A high-speed camera is placed in the direction of the fan-shaped liquid sheet plane to capture the morphological characteristics of the front of the spray liquid sheet. A second high-speed camera is placed on the side of the liquid sheet, and the structural feature information of the side view of the liquid sheet is recorded.

### 2.3 Spray visualization

The high-speed camera introduces fixed-mode noise (FPN) into the image through an image sensor. The light sensor captures the light, converts it into an electronic copy of the optical image, and then stores the video image. The high-speed camera is set to 2,000 frames/s. Under the pressure of 0.1–0.5 MPa, the morphological differences of the water atomization process and butachlor atomization process in the front view and side view directions of liquid sheet were captured.

The breakup length is defined as the distance of the pieces of the sheet rupture from the main sheet to form ligaments. The spray angle is determined as the angle formed by the boundary of the liquid sheet near the nozzle parallel to the flat fan sheet. The diffusion angle is determined as the angle formed by the boundary of liquid sheet in the direction of the side view. The image processing software Image-Pro Plus is used to measure the breakup length by calculating the pixel length in the picture. The angles are measured by the flexible two-point method also using the image processing software Image-Pro Plus (Wang, 2014).

### 2.4 Droplet size measurement

At the distance of 50 cm from the nozzle outlet (Xiao et al., 2018), the droplet size spectrum of water and butachlor was measured by a laser particle size analyzer when using a standard flat fan nozzle. The droplet size was measured by the laser particle size analyzer according to the “Fraunhofer” diffraction principle and the typical parallel optical path design. The laser particle size analyzer uses a photoelectric detector to collect signals such as scattered light intensity and energy, and then calculates and interprets according to the scattering principle to obtain particle size information. Under the same working conditions, the instrument carries out three measurements and produces three droplet size data. The most commonly used method to characterize the droplet size is through the volume median diameter D_V50_ (VMD). D_Vm_ is the diameter of the droplets with a cumulative distribution of m%. The relative span value (R) is the droplet distribution span/droplet spectrum width, which is an index to measure the droplet size distribution width (Dombrowski and Johns, 1963; Matthews et al., 2014). The test error is less than 3%. Under different pressure conditions of 0.1–0.5 MPa, the spraying distribution characteristics of droplet size was measured.

### 2.5 Dimensionless analysis

The dimensionless analysis method is used to analyze the obtained data. Dimensional analysis is an important method to explore the law of flow-through experiments, especially for those flow problems that are difficult to analyze theoretically. The dimensionless number generated by the fluid control equation can be used to describe the relevant physical changes in liquid sheet atomization (Altieri and Cryer, 2018). Within the accuracy range of this experiment, the viscosity has little effect on the droplet size (Kooij et al., 2018), and because of the significant correlation between surface tension and perforation mechanism in multiphase atomization mechanism, this study mainly focuses on the influence of surface tension and inertia force, that is, the Weber number on spray breakup mechanism (Yang et al., 2022). The surface tension of emulsion butachlor and water is measured by the CAM 101 (KSV, Finland) automatic tensiometer using the hanging drop method. The pendant drop method uses Laplace-Young fitting to fit the outline of the droplet to obtain surface tension. The Weber number is defined as (Tarnogrodzki, 1993):


(1) $$We=\frac{\rho v^{2}l}{\sigma }$$

where *ρ* is liquid density (kg/m^3^), *v* is characteristic velocity (m/s), *σ* is surface tension coefficient (N/m), and *l* is the characteristic length of the Weber number for the flat fan nozzle with an elliptical outlet, which is defined as:


(2) $$l=4*\frac{\text{A}}{X}$$

where A is the area of the flat fan nozzle outlet (m^2^) and X is the wetted perimeter (m).

Combining Equations (1) and (2) yields:


(3) $$We=\frac{4\text{A}\rho v^{2}}{\text{X}\sigma }$$

The Weber numbers of different surface tensions at different flow rates were measured to study the effect of the competition between surface tension and inertial force on the stability of liquid sheet and droplet breakup (Altieri et al., 2014). Using the dimensionless Weber number for dimensional analysis is helpful to find the functional relationship between physical quantities, especially for the complex fluid mechanics problem of agricultural spray.

## 3 Results

### 3.1 Morphological characteristics and evolution process of holes on liquid sheet

**Figure 2:**
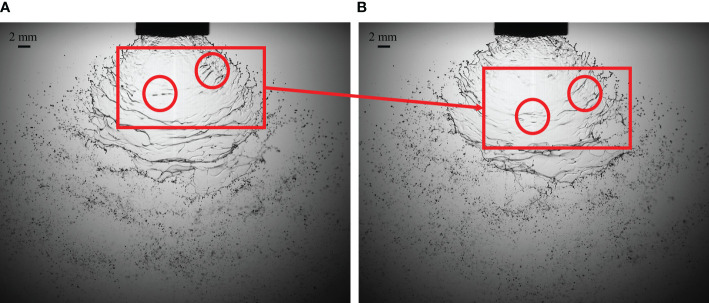
The features of wave structures in the water atomization process. The growth process of the typical liquid sheet unstable wave structures is marked by red circles. **(A)** the structural characteristics of the initial time of water atomization; **(B)** after 0.5 ms, the structure characteristics of water atomization.

**Figures 2A, B** show the atomization process of water in the direction of front view of liquid sheet. When the spray liquid is water, the liquid at the nozzle outlet deforms due to the shear force and fluctuates during the contraction process. The liquid sheet with wave is formed due to the disturbance when leaving the nozzle outlet. There is an obvious corrugated structure on the liquid sheet formed by water spray (Nadeem et al., 2018). As the disturbance on the liquid sheet increases, the wave structure on the liquid sheet gradually tears into a liquid ligament. The liquid ligament continues to break up into droplets due to Rayleigh–Taylor instability. These liquid ligaments are disintegrated into droplets by instability under the influence of surface tension (Gong et al., 2020; Wei et al., 2021).

**Figure 3:**
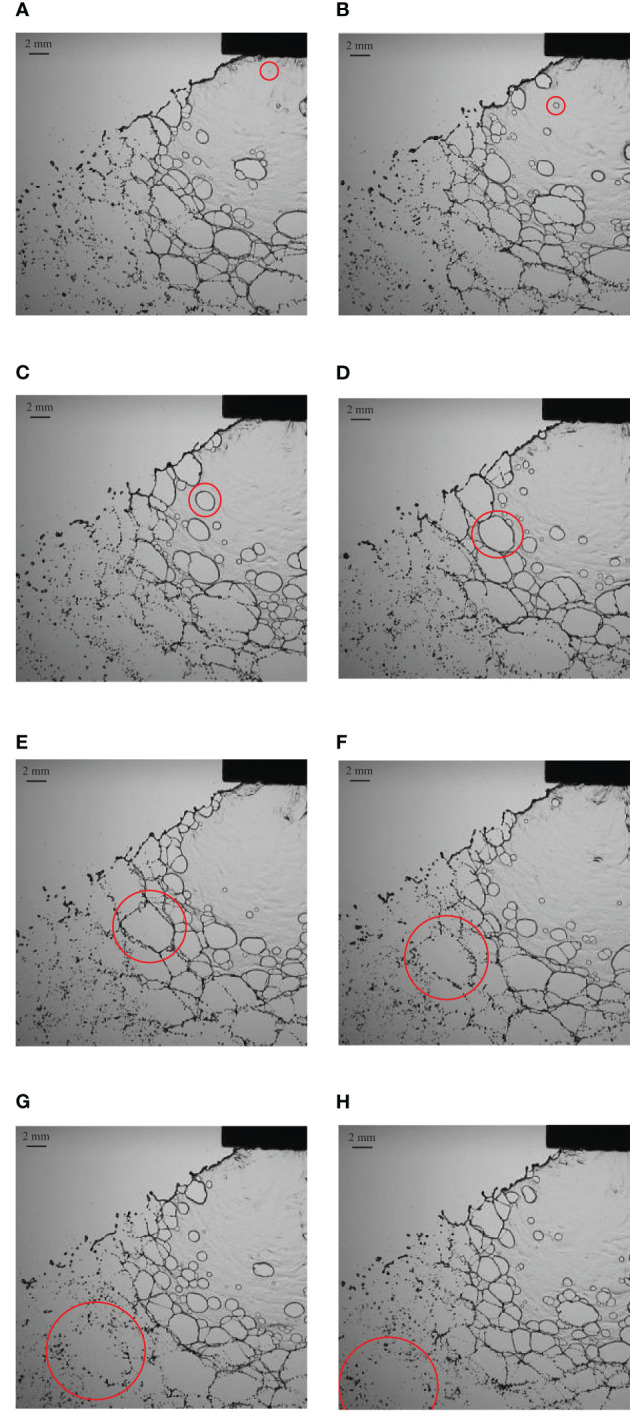
The evolution of hole structures in the emulsion butachlor atomization process. **(A)** 0 ms structure at the initial time; **(B)** 0.5ms structure; **(C)** 1 ms structure; **(D)** 1.5 ms structure; **(E)** 2 ms structure; **(F)** 2.5 ms structure; **(G)** 3 ms structure; **(H)** 3.5 ms structure.

**Figure 3** shows the formation and development of holes when the spray liquid is a hydrophobic butachlor solution. The liquid mass leaves the nozzle outlet to form the liquid sheet, and the pre-hole structure could be observed on the liquid sheet as shown in **Figure 3A**. The position where the pre-hole structure exists on the liquid sheet would form a broken hole, and the single broken hole and the surrounding broken holes gradually expand to form a net structure as shown in **Figure 3E**. The net structure continues to break up into droplets. The downward speed of the hole is basically maintained at a constant speed proximity to spray liquid jet velocity. When the pre-hole structures on the liquid sheet just forms a hole, the initial expansion speed of the hole increases slowly, then the expansion speed of the hole expansion increases, and finally the droplet is broken along the grid.

**Figure 4:**
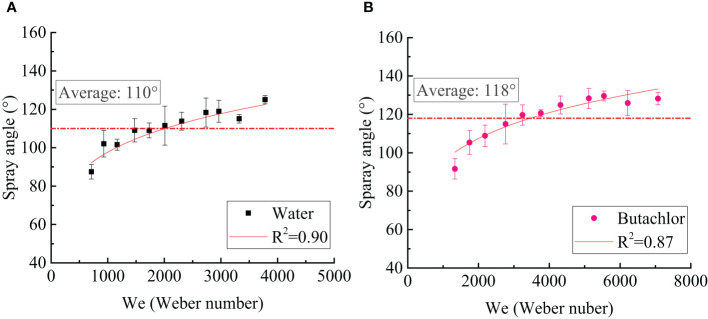
**(A)** Spray angle varies with the Weber number of water spray; **(B)** spray angle varies with the Weber number of butachlor spray.

The change of spray angle between water and butachlor is shown in **Figures 4A, B**. The spray angle indicates the change of spray swath and the rim disturbance of the liquid sheet. The spray angle of butachlor is 8° larger than that of water in the same operating pressure. The spray angle of water and butachlor has a similar change trend; that is, it increases slowly with the increase of the Weber number, and the increase gradually decreases. Sprays with pesticide butachlor lead to earlier breakup of the liquid sheet, droplet formation starting closer to the nozzle, and larger spray angles.

Combined with the dimensionless number analysis, the spray angle gradually increases with the increase of the Weber number. The spray angle remained at approximately 110° (Kooij et al., 2018). Quantitatively, the spray angle shows a power law dependence of the Weber number with a power of 0.17, which is suitable for all conditions tested here,


(4) $$\theta_{s}\propto We^{0.17}$$

where θ_s_ is the spray angle (°) and We is the Weber number (dimensionless).

**Figure 5:**
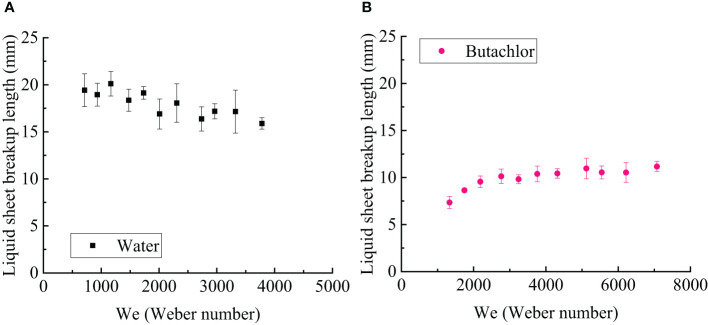
**(A)** Liquid sheet breakup length varies with the Weber number of water spray; **(B)** Liquid sheet breakup length varies with the Weber number of butachlor spray.

The liquid sheet breakup length of water and emulsion butachlor varies with the Weber number, as shown in **Figures 5A, B**. It could be seen that with the increase of the Weber number, the liquid sheet breakup length of water decreases slowly from 20 mm to approximately 16 mm, while the liquid sheet breakup length of butachlor increases slowly from approximately 7 mm to 12 mm, which changes less with the Weber number. It is obvious that the liquid sheet breakup length of butachlor is smaller than that of water.

With the presence of emulsion butachlor, the surface tension is different from the surface tension of water spray, which reduces the stability of the liquid sheet during spray, resulting in the formation of the liquid ligament closer to the nozzle, and making the atomization area of butachlor smaller. The liquid sheet breakup length of butachlor is shorter than that of water, which is consistent with the fact that the atomization breakup of butachlor occurs earlier (Ellis and Tuck, 1999; Xie et al., 2013).

**Figure 6:**
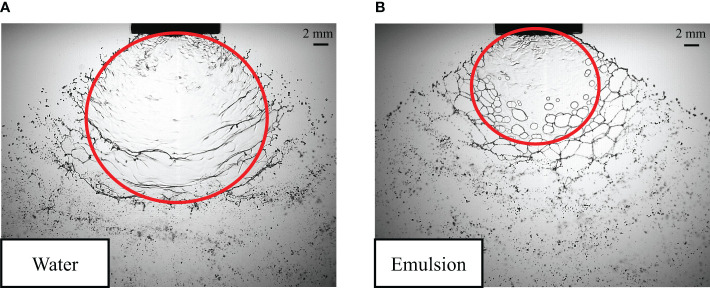
The liquid sheet area of water **(A)** and emulsion butachlor **(B)**.

As shown in **Figures 6A, B**, not only is the liquid sheet breakup length of butachlor less than that of water, but the liquid sheet expansion area of butachlor is significantly smaller than that of water as well. When the emulsion is used as the spray liquid, the liquid sheet fragmentation occurs in advance, while the spray angle increases (Gong et al., 2021). It indicates that emulsion butachlor advances the atomization process and changes the liquid sheet stability.

**Figure 7:**
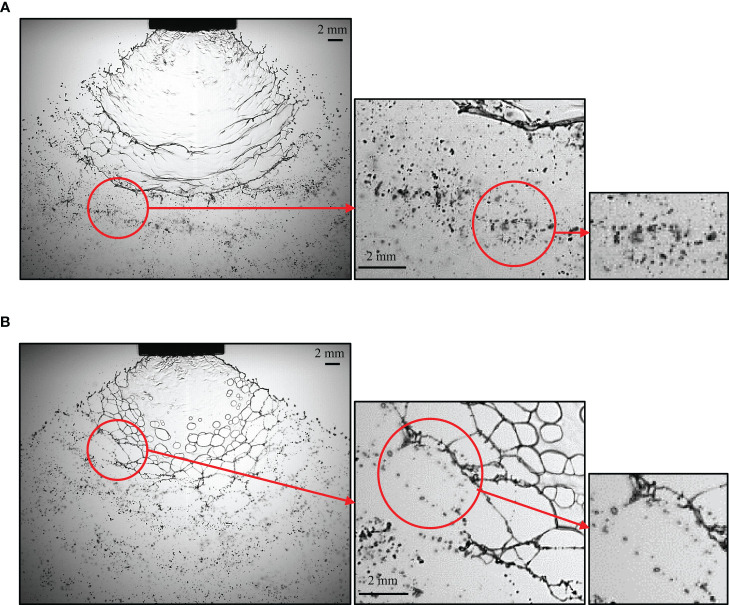
**(A)** Droplet morphology characteristics and formation of water spray; **(B)** Droplet morphology characteristics and formation of butachlor spray.

The droplets formed by the water spray and butachlor spray have different characteristics in morphology. It could be seen from **Figure 7A** that the liquid sheet formed by water atomization is broken into liquid ligaments due to oscillation disturbance, and the liquid ligaments destabilize and break up into droplets. The droplets are distributed from ligaments and gradually spread around (Qin et al., 2018). The droplet group formed by liquid ligament fragmentation also has the same wave-like distribution due to the disturbance. As shown in **Figure 7B**, when the spray liquid is emulsion butachlor, the morphology of the fragmentation process changes obviously, and holes appear on the liquid sheet to form the net structures. The fragmentation mechanism changes into perforation mechanism, and the droplets are also distributed around the net. It can be seen from the image that the droplet size formed by the water spray is smaller than that formed by the butachlor spray.

### 3.2 Disturbance and instability of liquid sheet

Water atomization and butachlor atomization also have different morphological characteristics from the side view of the liquid sheet. The fluctuation of the liquid sheet presents the stability of the liquid sheet.

**Figure 8:**
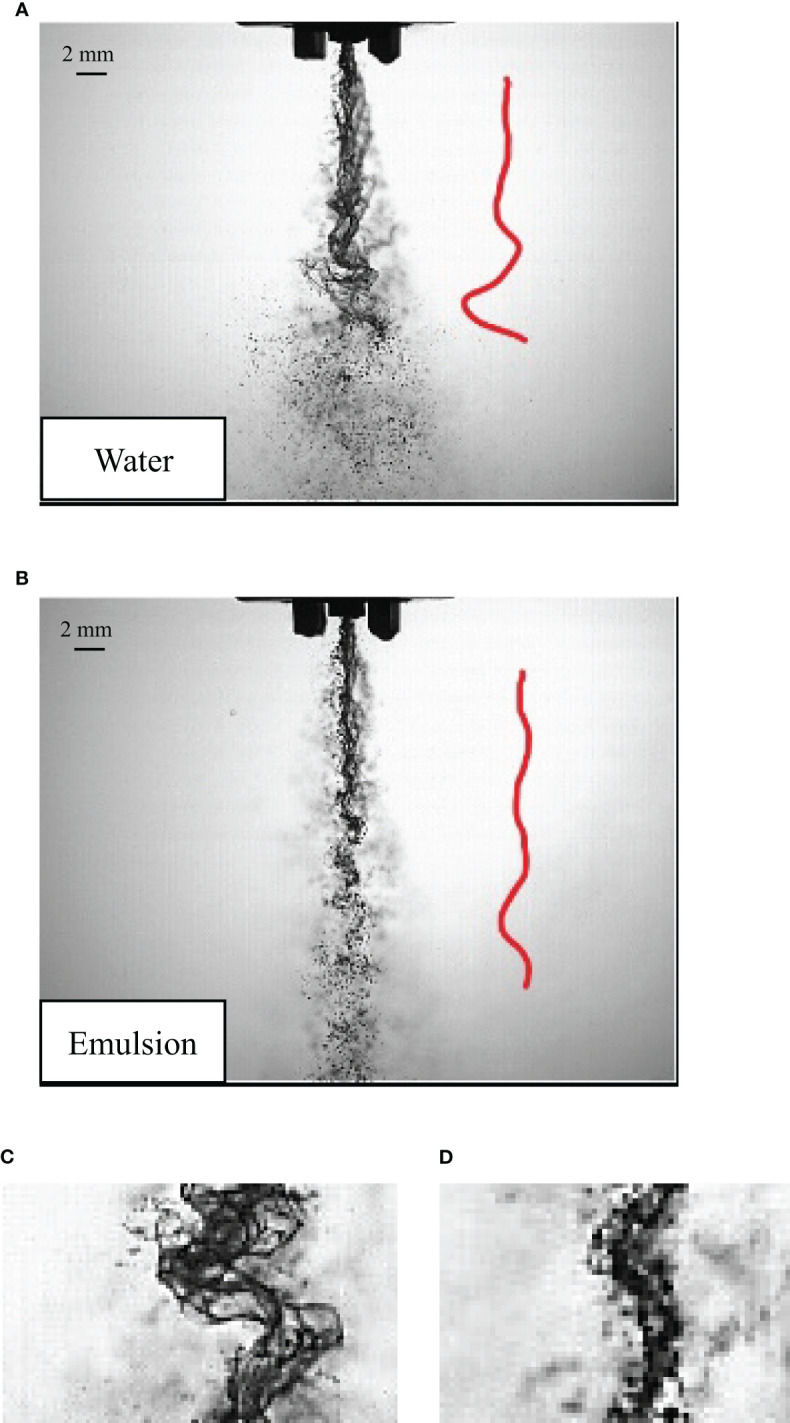
**(A)** The side view morphology of water spray; **(B)** The side view morphology of butachlor spray; **(C)** water morphology local; **(D)** butachlor morphology local.

It can be seen from **Figure 8A** that when the spray liquid is water, the disturbance fluctuation is more obvious and the fluctuation amplitude is larger. When the spray liquid is emulsion butachlor in **Figure 8B**, the fluctuation of the side is significantly reduced. The side view images of water atomization and butachlor atomization at the same position were intercepted respectively as shown in **Figures 8C, D**. It is obvious that the disturbance of the water spray is greater than that of butachlor. The appearance of droplets is earlier than that of the water spray in the side view image of butachlor.

**Figure 9:**
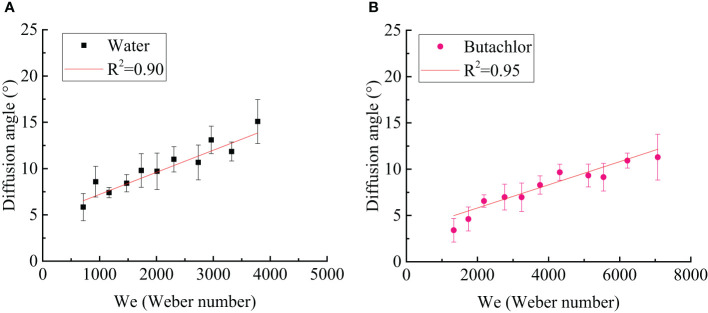
**(A)** Diffusion angle in water spray varies with the Weber number; **(B)** diffusion angle in butachlor spray varies with the Weber number.


As shown in **Figures 9A, B**, the diffusion angle increases with the increase of the Weber number. However, the increasing trend is weak. The diffusion angle of water is slightly larger than that of butachlor. This is consistent with the longer liquid sheet breakup length and more obvious swing of water. Moreover, all data collapse on one line of diffusion angle, i.e.,

(5) $$\theta_{d}\propto We$$

where θ_d_ is the diffusion angle (°) and We is the Weber number (dimensionless).

### 3.3 Effect of emulsion butachlor on atomization droplet size

Droplet size is one of the key parameters affecting spray quality. Different atomization mechanisms significantly affect the droplet size spectrum. As shown in **Table 1**, the droplet size Dv_50_ (volume median diameter) of butachlor is larger than that of water (Altieri and Cryer, 2018; Post and Hewitt, 2018). This is consistent with the liquid sheet structure characteristics captured by the high-speed camera. Fine size droplets are easy to drift. The addition of emulsion additives could effectively increase D_V50_ and decrease D_V10_, thereby reducing drift, and improving pesticide utilization (Altieri and Cryer, 2018).

**Table 1:** Dv_50_, Dv_10_, Dv_90_, and relative span (uniformity of droplets size) for sprays produced with water and emulsion butachlor.

Spray liquid		0.1 MPa	0.2 MPa	0.3 MPa	0.4 MPa	0.5 MPa
Water	Relative Span	1.165	1.216	1.187	1.370	1.467
	Dv_50_ (μm)	229.563	178.668	156.067	146.817	138.365
	Dv_10_ (μm)	122.207	85.743	76.636	68.494	63.024
	Dv_90_ (μm)	389.548	302.935	261.809	269.643	265.973
Oil-based emulsion butachlor	Relative Span	1.067	1.2387	1.172	1.183	1.213
	Dv_50_ (μm)	387.474	234.55	194.57	178.46	165.608
	Dv_10_ (μm)	201.702	128.817	98.989	86.644	75.5
	Dv_90_ (μm)	615.113	419.26	326.967	297.812	276.434

The influence of spray process on the formation of droplets is further analyzed by analyzing the relative span. The relative span is a measure of droplet size distribution. The smaller the relative span, the narrower the droplet size spectrum, and the more consistent the droplet size. The droplet size decreases with the increase of spray pressure, regardless of spray type (water or butachlor), while the relative span increases with the increase of pressure. Butachlor emulsion spray not only increased the droplet size but also improved the uniformity of droplet size distribution. At 0.3 MPa, D_V50_ increased 25%, and the span decreased from 1.187 to 1.172. This may be attributed to the fact that the spray of emulsion butachlor changes the formation process of droplets and weakens the disturbance of the liquid sheet. The droplet size generated from the hole structure is more uniform than that generated from the surface wave structures. This result helps to improve the quality of sprays and reduces pesticide waste by adjusting pesticide formulations.

## 4 Discussion

Pesticide spraying is the primary means of controlling insects and weeds. The use of pesticides has a significant impact on crop yields and the health of surrounding ecosystems and workers (Paudel et al., 2020). However, the atomization mechanism of pesticide spraying is complex, and the relevant theoretical research is insufficient (Hilz et al., 2012). Taking water and emulsion concentrate pesticides as the research object aimed to deeply analyze the influence of emulsion concentrate pesticide atomization on atomized droplet size based on the study of the characteristics, development, and fragmentation process of the liquid sheet structure and the evolution process of fragmentation. The characteristics of the spray liquid sheet structure reflect the liquid sheet breakup dynamics (Li et al., 2020).

When the water is atomized, the structural characteristics of the liquid sheet are mainly the surface wave structures of continuous disturbance. This is because the main cause of the instability of the liquid sheet is the velocity difference between the liquid phase and the surrounding air phase. The existence of velocity difference leads to the generation of surface wave structures. With the development of surface wave, the liquid sheet breaks up to form droplets. Emulsion pesticide atomization has completely different structural characteristics; that is, it has completely different fragmentation mechanical properties. The presence of emulsion components leads to the appearance of hole structures on the liquid sheet (Goual et al., 2020; Siddharth, 2021). The existence of holes changes the instability process of liquid sheet and the formation of droplets (Li et al., 2021).

The development of liquid sheet is affected by surface tension and inertial force. Therefore, this study quantitatively studied the relationship between the Weber number and liquid sheet structure characteristics, which is used to characterize the process of liquid sheet instability and droplet formation. We found that the perforation mechanism slightly increased the spray angle (Kim et al., 2021). Under the same surface tension, only the spray velocity is changed; that is, only the inertia force is changed. The spray angle of either mechanism shows the same trend with the Weber number; that is, it shows a power law relationship with the Weber number. When the inertia force is the same, the smaller the surface tension, the larger the spray angle. The size of the liquid sheet breakup length reflects the order of liquid sheet instability. The liquid sheet breakup length of the emulsion butachlor is smaller than that of water, which proves that the emulsion leads to the early breakage of the liquid sheet. Compared with water atomization, pesticide atomization is less affected by inertial force. The change trend of the diffusion angle with the Weber number also confirms each other. In summary, it leads to a larger droplet size and a more consistent droplet size spectrum of the emulsion pesticide butachlor atomization.

Emulsion pesticide sprays involve the complex multi-phase flow of water, oil, gas, and solid (Cryer et al., 2021). Their flow structure and flow characteristics are more complicated than those of pure water. Correlation studies involve a challenging scientific exploration and expansion of the theoretical system of single-phase fluid atomization. The purpose of reducing spray drift, improving atomization quality, and improving the effective utilization rate of pesticides could be achieved by preparing appropriate liquid and matching appropriate spray conditions to control spray stability, and then controlling the size and speed of droplets (Makhnenko et al., 2021).

## 5 Conclusions

In this paper, the atomization process through a standard flat fan nozzle of water and butachlor emulsion was visualized using a high-speed camera. By means of experimental research and dimensionless analysis, the differences of the atomization process and droplet size spectrum between emulsion pesticide spray and water spray were compared:

(1) The spray angle of butachlor atomization is larger than that of water atomization. The spray angle shows a power law dependence of the Weber number with a power of 0.17 for all conditions tested here. However, the liquid sheet breakup length of emulsion butachlor is smaller than that of water.
(2) In the process of water atomization, the side view of the liquid sheet is a large waveform disturbance, while that of the butachlor atomization is a small waveform disturbance. In the meantime, the diffusion angle of water is also greater than that of butachlor.
(3) The atomization process and mechanism of water and emulsion butachlor are different. During water atomization, the fragmentation process in the direction of the fan-shaped liquid sheet plane shows that the liquid mass leaves the nozzle to form a liquid sheet. Wave structures are shown on the liquid sheet due to the disturbance. The unstable liquid sheet breaks up into liquid ligaments, and the liquid ligaments break up into droplets. The presence of butachlor led to the formation of pre-holes on the liquid sheet. The pre-holes developed into holes, and the holes formed a net structure and finally break up into droplets. The size of the droplets formed by the atomization of butachlor is larger than that of water atomization due to the change of fragmentation mechanisms. The presence of emulsion improves the uniformity of spray droplet size distribution. The increase in droplet size is of great significance for spray anti-drift.

## Data availability statement

The original contributions presented in the study are included in the article/supplementary material. Further inquiries can be directed to the corresponding author.

## Author contributions

WY: Conceptualization, Data curation, Formal Analysis, Investigation, Methodology, Software, Validation, Visualization, Writing – original draft, Writing – review & editing. WZ: Conceptualization, Data curation, Formal Analysis, Investigation, Methodology, Software, Validation, Visualization, Writing – original draft, Writing – review & editing. WJ: Conceptualization, Funding acquisition, Project administration, Resources, Supervision, Writing – review & editing. MO: Conceptualization, Funding acquisition, Project administration, Resources, Supervision, Writing – review & editing. XD: Conceptualization, Project administration, Resources, Supervision, Writing – review & editing. TZ: Writing – review & editing. SD: Writing – review & editing. LJ: Writing – review & editing. XW: Writing – review & editing.
